# Metformin improves age-related visual cortex dysfunction in mice by reducing noise correlation in the primary visual cortex

**DOI:** 10.3389/fnagi.2025.1572653

**Published:** 2025-06-26

**Authors:** Xiaoming Liu, Yifeng Zhou, Jiachen Liu, Guangwei Xu

**Affiliations:** ^1^CAS Key Laboratory of Brain Function and Diseases, Division of Life Sciences and Medicine, University of Science and Technology of China, Hefei, Anhui, China; ^2^Vision Research Laboratory, Division of Life Sciences and Medicine, University of Science and Technology of China, Hefei, Anhui, China; ^3^Anhui Medical College, Hefei, Anhui, China

**Keywords:** aging, metformin, noise correlation, inhibitory neurons, primary visual cortex

## Abstract

**Introduction:**

Age-related decline in visual processing has been observed in association with reduced orientation selectivity and decreased signal-to-noise ratios in the primary visual cortex (V1). Elevated noise correlations between neurons are associated with impaired visual discrimination in aging; however, less is known about therapeutic interventions that could preserve visual cortical function during aging. In this study, we investigated the effects of metformin treatment on age-related changes in visual processing and neuronal correlations in V1.

**Methods:**

We conducted *in vivo* electrophysiological recordings to investigate whether 3 weeks of acute gavage with metformin improves visual processing in 12-month-old mice compared to 8-week-old mice by modulating neural noise in the V1, and used western blot analysis to investigate the molecular mechanism of the effect of metformin.

**Results:**

*In vivo* electrophysiological recordings revealed that aging led to V1 neuronal hyperactivity, accompanied by reduced orientation selectivity, a decreased signal-to-noise ratio, and increased response variability. Notably, aged mice exhibited increased noise correlation, response covariance, and population variability. Analysis of fast-spiking interneurons revealed impaired noise suppression in the inhibitory circuits of aged mice. Daily metformin treatment reversed these age-related alterations by improving fast-spiking neuron-mediated decorrelation and reducing noise correlation. Mechanistically, metformin upregulated the protein expression levels of glutamic acid decarboxylase 67 and gephyrin, key components of inhibitory synapses, suggesting that metformin enhances visual processing by strengthening inhibitory signaling and reducing the correlated variability in the V1.

**Discussion:**

Metformin treatment effectively ameliorated these deficits through enhanced GABAergic signaling; however, the broader therapeutic mechanisms across sensory systems remain unclear. In this study, we demonstrate that metformin preserves visual function by restoring excitatory-inhibitory balance, suggesting a promising approach for age-related sensory decline.

## Introduction

Aging is widely recognized as a risk factor for ocular diseases. However, even in the absence of overt ocular pathologies, the age-related decline in visual processing can substantially affect the daily activities and quality of life of older adults. Among the key contributors to this decline is diminished information-processing capacity within the visual cortex (Andersen, 2012). Electrophysiological recordings of aging macaques have shown a reduction in the orientation selectivity of primary visual cortex (V1) neurons (Schmolesky et al., 2000; Yu et al., 2006); studies on aged cats reported similar findings (Hua et al., 2006). Neuronal orientation discrimination relies on precise inhibitory control and some studies have indicated that increased cortical noise in older animals may exacerbate orientation tuning deficits (Delahunt et al., 2008). Notably, the administration of γ-aminobutyric acid (GABA) or its agonists can restore orientation tuning in aging animals (Leventhal et al., 2003), potentially by harnessing the capacity of the GABAergic system to regulate cortical noise levels. These findings strongly suggest that dysregulation of the inhibitory system is a critical driver of age-related visual impairment.

The GABAergic system serves as a vital regulator of excitatory–inhibitory (*E*/*I*) balance in the cortex, ensuring that neuronal representations remain selective and resistant to interference (Kepecs and Fishell, 2014; Tremblay et al., 2016). Impaired GABA production or dysfunction of GABAergic receptors can lead to neuronal hyperexcitability and desynchronized activity, resulting in compromised receptive field properties and reduced sensory perception (Churchland et al., 2010; Moreno-Bote et al., 2014). The age-related reduction in cortical inhibition has been linked to diminished receptive field specificity, lower signal-to-noise ratios, and weaker orientation tuning (Betts et al., 2007; Zhang et al., 2008). Our previous study on aged macaques demonstrated that the functional decline of the inhibitory system, reflected in changes to GABAergic neurotransmission, was closely associated with deficits in V1 neuronal responses (Wang et al., 2019). Collectively, these observations indicate that compromised GABAergic inhibition is a fundamental contributor to age-related visual deterioration.

Therapeutic strategies that increase GABAergic function may therefore mitigate visual decline. Metformin, a first-line oral medication for the treatment of type 2 diabetes mellitus (T2DM), has been shown to have neuroprotective effects in studies of various neurological disorders (Paudel et al., 2020; Sanati et al., 2022). It readily crosses the blood–brain barrier and accumulates in the central nervous system (Nath et al., 2009). It has also been reported to upregulate GABA_A_ receptor-related proteins, facilitate receptor membrane insertion, and increase miniature inhibitory postsynaptic currents in cultured rat hippocampal neurons (Fan et al., 2019). In diabetic epilepsy rats, metformin treatment restored abnormal glutamate–GABA levels (Mohamed et al., 2020), highlighting its potential to modulate the *E*/*I* balance. Moreover, metformin has been shown to extend lifespan and delay aging in several organisms (Anisimov et al., 2011, 2010, 2008, 2005; Onken and Driscoll, 2010). Despite these findings, whether metformin can slow or reverse the decline in age-related visual processing remains unclear.

In the present study, we tested the hypothesis that metformin improves visual cortical function in older animals through the enhancement of GABAergic signaling, by examining the tuning properties, response properties of V1 neurons and neuronal ensemble coding properties in aged mice. We further explored the molecular mechanisms underlying these changes. Metformin not only reduced excessive cortical activity but also improved orientation selectivity in aged mice, potentially by increasing GABA production and receptor function.

Given the extensive clinical use of metformin and its well-established safety profile for the treatment of T2DM, it is a promising candidate for the treatment of age-related visual decline. Metformin may improve inhibitory signaling within the visual cortex, contributing to the preservation or restoration of sensory processing in older individuals. These insights also underscore the broader value of targeting GABAergic modulation to counteract age-associated neurophysiological deficits, highlighting a potential framework for future research on neuroprotective interventions and healthy aging processes.

## Methods

### Animals

A total of 20 male C57BL/6J mice were used in electrophysiological experiments, six 8-week-old mice in the young group, five 12-month-old mice in the aged group, and seven 12-month-old mice in the metformin-treated (met) group. A total of 41 male C57BL/6J mice were used for western blot analysis, 13 in the young group, and 14 in each of the aged and met groups. Mice were obtained from Charles River (Wilmington, MA, USA) and housed in cages with plenty of water and standard mouse chow under a 12 h light/dark cycle at a stable temperature of 23–25°C. All experiments were designed to minimize the use of mice and alleviate their suffering. All animal protocols were approved by the Animal Care and Use Committee of the University of Science and Technology of China (approval number USTCACUC25020123093).

### Metformin treatment

Met group mice received 250 mg/kg metformin hydrochloride per day by oral gavage (Foretz et al., 2014; Sanchis et al., 2019). Metformin hydrochloride powder (D150959-5G; Sigma-Aldrich, St. Louis, MO, USA) was dissolved in water to a concentration of 25 mg/mL; each 20 g mouse received 0.2 mL of this solution. This dose corresponds to a human dose of 1,200 mg/day, which is the average recommended dose for patients with T2DM (range, 500–2,000 mg/day) (Oliveira et al., 2021). After gavage, mice were returned to their cages and allowed free access to water. Electrophysiological recordings were performed after 3 weeks of daily gavage. During the electrophysiological recordings of one mouse, the remaining mice continued to receive metformin treatment.

### Stereotaxic surgery

Anesthesia was induced by an intraperitoneal injection of 250 mg/kg tribromoethanol mixed with 500 mg/kg tert-amyl alcohol and maintained using 1–2% isoflurane. Mice were positioned on a stereotactic frame with ear bars and a mouth retractor. After subcutaneous injection of 10 mg/kg lidocaine hydrochloride mixed with 3 mg/kg bupivacaine hydrochloride for local anesthesia, a portion of the scalp over the skull was removed and the cranial fascia was removed with 3% hydrogen peroxide. Vetbond tissue adhesive (3M, Saint Paul, MN, USA) was applied to protect and repair the scalp tissue. The surface of the skull over the V1 was roughened with a cranial drill, and the V1 was marked (anterior-posterior: −3.5 mm; medial-lateral: ±2.5 mm). A titanium alloy headplate was then attached to the skull with a denture base resin and denture water. After solidification, the mice were returned to their cages for 1 week of recovery.

### Visual stimuli

Visual stimuli were generated using the Psychophysics Toolbox-3 extension in MATLAB (MathWorks, Natick, MA, USA) and were displayed on an LCD screen (resolution: 1,920 × 1,080 pixels; refresh rate: 144 Hz; average brightness: 45.2 cd/m^2^; PG259; ASUS, Taipei, Taiwan); screen brightness was gamma-corrected non-linearly. The display was positioned 30 cm from the eyes of the mouse at an angle of 45° relative to its body axis. The stimuli consisted of motion sinusoidal gratings with directions ranging from 0° to 360° in 30° steps, as well as blank gray-screen stimuli without gratings, and the average brightness of the blank stimulus is consistent with that of the grating stimulus. Blank gray-screens are also presented between trials. The grating motion direction was perpendicular to its orientation. Each visual stimulus was presented for 1 s, with the contrast set at 95% of the maximum. Each stimulus or control presentation was defined as a trial, and the total number of trials with different grating orientations and controls was considered as one sweep. In each sweep, the gratings and control stimuli of different orientations were presented in a pseudorandom order. Ten sweeps were recorded in each experiment.

The spatial and temporal frequencies, as well as grating orientations, that elicited maximal responses from the largest number of neurons under the electrode recording conditions were selected. These conditions were then used to test the neuronal responses to gratings at different orientations. The optimal orientation of a neuron is the one that elicits the highest firing rate.

### Electrophysiological recordings

The titanium-alloy mouse headplate was fixed to a U-shaped mounting device using screws to ensure immobilization of the head but free movement of the body and limbs. After fixation and adaptation, a hole ~0.5 mm in diameter was drilled in the skull above the V1 according to the previously placed marker (anterior-posterior: −3.5 mm; medial-lateral: ±2.5 mm), exposing the brain tissue. Neuronal responses in the 0–1,000 μm depth range of the V1 region were recorded using a 1 × 32 linear silicon electrode array (spacing, 25–50 μm; NeuroNexus Technologies, Ann Arbor, MI, USA). All neuronal signals were filtered (750–4 kHz), amplified using a preamplifier (cutoff frequency, 10 kHz; Blackrock Neurotech, Salt Lake City, UT, USA), and digitized using a neural signal processing system (sampling frequency, 30 kHz; 16 bits; Blackrock Neurotech). The data were saved for offline clustering analysis using Offline Sorter software version 3.3.5.0 (Plexon, Inc., Dallas, TX, USA). Principal component analysis was used to calculate the waveform characteristics of neuronal firing. By analyzing the clustering patterns in the principal component analysis (PCA) space, the signals recorded by the electrode channels were separated from the background noise and identified as single units, which were defined as the response signals of single neurons (single-unit activity, SUA).

### Electrophysiological data analysis

The electrophysiological recordings were analyzed using MATLAB software. The average response intensities of neurons during blank and grating stimulations were defined as the spontaneous and stimulus-evoked responses, respectively.

The orientation selectivity index (OSI) is typically measured as the difference between the responses to the optimal orientation and the orthogonal orientation, divided by the sum of the responses to the optimal and orthogonal orientation.


(1) $$\begin{array}{l} OSI=\left(\frac{{R_{optimal}-R}_{orthogonal\;}}{R_{optimal}+R_{orthogonal}}\right)\times 100\% \end{array}$$

Here, *R*_optimal_ represents the average response of the neuron to the optimal orientation, calculated as the mean of the responses in the optimal direction and the null direction. Meanwhile, *R*_orthogonal_ denotes the average response of the neuron to the orientation that is orthogonal to the optimal orientation. Specifically, *R*_orthogonal+_ and *R*_orthogonal−_ represent the responses of the neuron to the orthogonal orientation moving in two opposite directions. *R*_orthogonal_ is obtained by averaging the responses in the orthogonal+ direction and the orthogonal– direction. These orientations were derived from the fitted curves using the double von Mises function.


(2) $$\begin{array}{l} f\left(X|\theta_{1},\theta_{2},k_{1},k_{2},\mu \right)=\frac{e^{k_{1}cos\left(x-\theta_{1}\right)}}{2\pi I_{0}\left(k_{1}\right)}+\frac{e^{k_{2}cos\left(x-\theta_{2}\right)}}{2\pi I_{0}\left(k_{2}\right)}+\mu \end{array}$$

Here, *X* is the average response of a neuron to a stimulus in a particular orientation; θ1 and θ2 are the optimal stimulus orientation and its opposite orientation, respectively, and usually differ by π in radians; *k*_1_ and *k*_2_ are the wave width parameters, which represent the bandwidths of the neuronal orientation tuning curve; *I*_0_ is the 0th-order Bessel function; and μ is the baseline response of the neuron. After obtaining the fitted tuning curve, neurons with a goodness of fit >0.6 were selected to calculate the OSI.

To quantify the ability of neurons to discriminate stimulus-evoked activity from spontaneous activity, we computed the signal-to-noise ratio (SNR) using a receiver operating characteristic (ROC) analysis. In this approach, the distribution of neuronal responses during presentation of the optimal orientation (“evoked”) was compared to the distribution during the gray screen period (“spontaneous”).

For each neuron, we constructed an ROC curve by systematically varying a threshold across the full range of observed response values. At each threshold, we calculated the true positive rate (TPR)—the proportion of stimulus-evoked response bins exceeding the threshold—and the false positive rate (FPR)—the proportion of spontaneous response bins exceeding the threshold. By sweeping the threshold from the minimum to the maximum response value, we generated a series of (FPR, TPR) pairs, which together form the ROC curve.

The signal-to-noise ratio was then defined as the area under the ROC curve (AUC), which quantifies the overall discriminability between the two response distributions. An AUC of 0.5 indicates chance-level discrimination, whereas an AUC of 1.0 corresponds to perfect separation between evoked and spontaneous responses. Importantly, this ROC-based SNR is threshold-independent, as it systematically considers all possible thresholds rather than relying on an arbitrary or fixed cut-off.


$$\begin{array}{l} SNR=AUC\left(x,y\right) \end{array}$$

Here, AUC represents the area under the curve formed by the intersection of the two curves; x represents the curve obtained by calculating the proportion of bins with spontaneous neuronal responses above the threshold; and y represents the curve obtained by calculating the proportion of bins with neuronal responses at the optimal orientation above the threshold.

Neuronal response variability refers to fluctuations in the neuronal response intensity when the same grating stimulus trial is repeated across different sweeps. The smaller the fluctuation, the fewer the signal changes during transmission, and the higher the fidelity. The Fano factor (FF), defined as the ratio of the variance to the mean of the neuronal response, was used to measure the neuronal response variability.


$$\begin{array}{l} FF=\frac{\sigma^{2}}{R_{means}} \end{array}$$

Here, σ^2^ is the variance of the neuronal response across different trials; and *R*_means_ is the average neuronal response across different trials.

In a local neural network, neighboring neurons receive shared sensory inputs, leading to shared response variability (Azeredo da Silveira and Rieke, 2021). The degree to which this variability is shared is known as noise correlation. Noise correlation is defined as the Pearson correlation coefficient of the response variability between two neurons to the same repeated stimulus.


$$\begin{array}{l} r_{sc}=\frac{E\left(N_{1}\cdot N_{2}\right)-E\left(N_{1}\right)\cdot E\left(N_{2}\right)}{\sigma_{N_{1}}\cdot \sigma_{N_{2}}} \end{array}$$

Here, E is the expectation; σ is the standard deviation; and *N*1 and *N*2 are two sets of neuronal response fluctuations. The element of the set is the neuronal response fluctuation amplitude z under each trial.


$$\begin{array}{l} z=\frac{R_{trial}-E\left(R\right)}{\sigma } \end{array}$$

Here, *R*_trial_ is the neuronal response at each trial; E is the mean of the neuronal response; and σ is the standard deviation of the neuronal response.

Covariance was calculated to measure the population response variability of neurons when the same visual stimulus trial was presented in different sweeps.


$$\begin{array}{l} cov\left(N_{1},N_{2}\right)=E\left(N_{1}\cdot N_{2}\right)-E\left(N_{1}\right)\cdot E\left(N_{2}\right) \end{array}$$

Here *E* is the expectation; and *N*1 and *N*2 are two sets of spike counts for cells 1 and 2, respectively.

In this study, mutual information (MI) was used as a theoretical measure of the amount of information that is carried and encoded by a population of neurons. The Information Decomposition Toolbox was employed to evaluate the MI, with the Panzeri–Treves correction method used to account for biases in MI estimates caused by the limited sampling of neuronal responses (Panzeri et al., 2007; Panzeri and Treves, 1996). MI is defined as the difference between the response and noise entropies.


$$\begin{array}{l} MI\left(S;R\right)=H\left(R\right)-H(R|S)\\ \;H\left(R\right)=-\sum_{r}P\left(r\right)\log_{2}P(r|s)\\ \;H\left(R|S\right)=-\sum_{s=1}^{N_{s}}P(s)\sum_{r}P(r|s)\log_{2}P(r|s) \end{array}$$

Here, *H*(*R*) is the response entropy; *H*(*R*|*S*) is the noise entropy; *P*(*r*) is the probability that the neuronal response is r; and *P*(*r*|*s*) is the conditional probability that the response of s to a given stimulus is *r*.

The noise correlation between excitatory and inhibitory neurons was examined to calculate the difference between the noise correlations of evoked and spontaneous responses (Middleton et al., 2012; Renart et al., 2010; Tetzlaff et al., 2012). A positive difference indicated the absence of a decorrelation effect between these neurons; a value of zero was assigned in such cases. Conversely, a negative difference suggested the presence of a decorrelation effect; the larger the absolute value of the negative difference, the stronger the decorrelation effect.

### Western blotting

The visual cortical tissues of mice in each group were separated. For each sample, a lysis buffer was added at a concentration of 10 μL/mg tissue (ratio immunoprecipitation assay buffer, BL504A; Biosharp, Hefei, China). Samples were thoroughly homogenized to ensure complete lysis and protein extraction. After the addition of phosphatase and protease inhibitors at a 98:1:1 volume ratio, the tissues were lysed on ice for 1 h. The mixture was then centrifuged at 12,000 rpm at 4°C for 15 min to collect the supernatant. The protein concentration was quantified using a bicinchoninic acid assay kit (Biosharp) and adjusted to ensure equivalent concentrations across different samples. The samples were then aliquoted and stored. Prior to electrophoresis, an equal volume of 2 × loading buffer was added and the mixture was heated in a boiling water bath for 10 min. The proteins were separated using SDS-PAGE at a constant voltage of 80 V. Following separation, proteins were transferred to a methanol-activated polyvinylidene fluoride membrane at a constant current of 200 mA. Next, the membrane was blocked at room temperature for 1 h with 5% bovine serum albumin (BSA) and then incubated with primary antibody (prepared in 1% BSA) overnight at 4°C. After washing with PBST to remove the unbound primary antibody, the membrane was incubated with secondary antibody at room temperature for 1 h and then washed again with PBST. Enhanced chemiluminescence was detected using a molecular imaging system (LAS4000; GE HealthCare, Chicago, IL, USA) and protein expression levels were quantified using ImageJ software version 1.4.3 (National Institutes of Health, Bethesda, Maryland, USA). The grayscale values of the target proteins were analyzed and normalized to those of GAPDH before conducting statistical comparisons.

The primary antibodies used in this study were: anti-GABA_A_Rα1 (1:1,000; AB252430; Abcam, Cambridge, UK), anti-GABA_A_Rγ2 (1:1,000; AB288564; Abcam), anti-glutamate decarboxylase (GAD)65 (1:1,000; 21760-1-AP; Proteintech Group, Inc., Rosemont, IL, USA), anti-GAD67 (1:1,000; AB26116; Abcam), anti-Gephyrin (1:1,000; 14304; Cell Signaling), and anti-GAPDH (1:1,000; AB8245; Abcam). The secondary antibodies used were: goat anti-mouse (1:5,000; BL001A; Biosharp) and goat anti-rabbit (1:5,000; BL003A; Biosharp).

## Statistical analysis

Data are presented in two distinct formats: For violin plots, data are presented as the median ± interquartile range, while for line graphs and bar graphs, data are presented as the mean ± standard error of the mean. Mann–Whitney U-test was used to test for significance. Two-way analysis of variance (ANOVA) was used to establish differences in *r*_sc_ and mutual information data between the two groups. Effect sizes were calculated to complement the *p*-values and provide additional information about the magnitude of differences. For parametric tests, Cohen's d was used to quantify effect size, while for non-parametric tests, Cliff's Delta was calculated (Meissel and Yao, 2024). *p* < 0.05 was considered to indicate a statistically significant difference.

## Results

**Figure 1:**
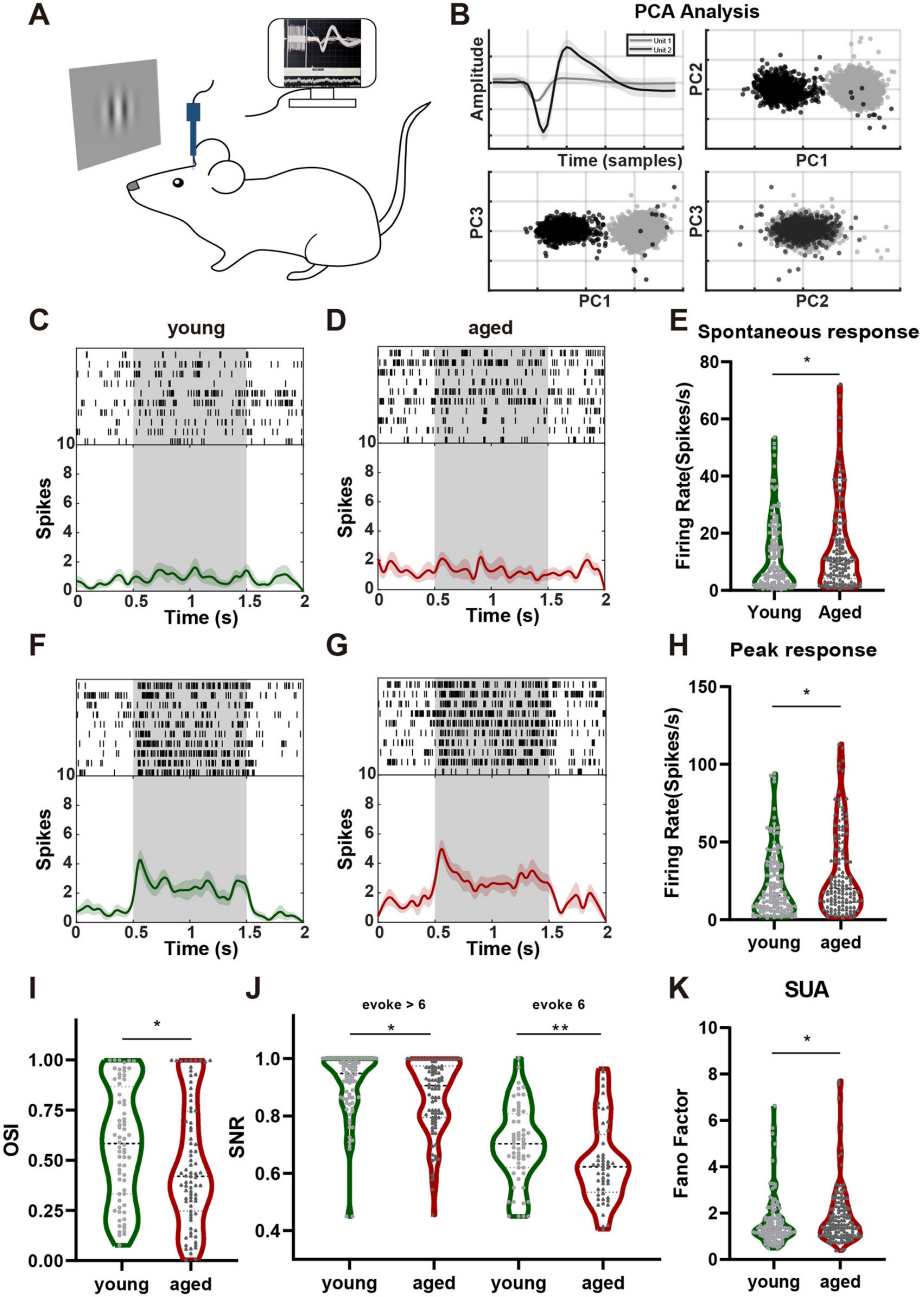
Aging affects the function of V1 neurons. **(A)** Schematic diagram of the electrophysiological recordings. **(B)** Example of separating a spike from baseline. **(C)** Example of a spontaneous neuronal response in the young group. **(D)** Example of a spontaneous neuronal response in the aged group. The gray parts represent the stimulus presentation period, and the upper parts show the response of the neuron to the gray screen under 10 different trials. Each vertical bar represents one delivery. The curves in the lower parts represent the average distribution of the bins, with the green and red shaded parts representing the standard deviations. **(E)** The spontaneous response was higher in the aged group than in the young group (Cliff's δ = −0.1438). **(F)**. Example of a neuronal response to the optimal orientation stimulus in the young group. **(G)** Example of a neuronal response to the optimal orientation stimulus in the aged group (Cliff's δ = −0.1651). **(H)** The peak response was higher in the aged group than in the young group. **(I)** The orientation selectivity index was lower in the aged group than in the young group (Cliff's δ = 0.1818). **(J)**. Differences in the SNR of neurons with firing rates >6 (left, Cliff's δ = 0.1836) and ≤ 6 (right, Cliff's δ = 0.2921). **(K)** The FF was higher in the aged group than in the young group (Cliff's δ = −0.1614). Data are presented as median and interquartile range in violin plots. **p < 0.01, *p < 0.05. FF, Fano factor; SNR, signal-to-noise ratio; V1, primary visual cortex.

Electrophysiological recordings were conducted in the V1 as shown in Figure 1A. A total of 136 neurons were recorded and isolated from the six 8-week-old mice; 24, 19, 23, 19, 27, and 24 from each mouse. A total of 140 neurons were recorded from the five 12-month-old mice; 32, 32, 22, 35, and 19 from each mouse (Figure 1B). The response characteristics of these neurons were analyzed after excluding outliers, which were identified based on the 3σ principle.

### Aging affects the function of V1 neurons

The response intensity of V1 neurons in the aged mice was significantly higher than that in young mice. The spontaneous and maximum evoked responses of neurons in both the young and aged groups are shown in Figure 1. The firing rate of representative neurons were determined during a 2-s window (from 0.5 s before to 0.5 s after stimulation) across 10 trials, in which gray screen blank stimulation and preferred visual stimulation were repeatedly presented. Both young and aged mice exhibited spontaneous neuronal firing in response to the gray screen, but this response was significantly greater in aged mice than in young mice (Figures 1C–E; Mann–Whitney U-test, *n* = 136, 140; median = 6.739, 10.45; IQR = 16.98, 16.8; *p* = 0.0391). Upon presentation of the preferred visual grating stimulus, both groups exhibited clear evoked responses. However, the response intensity was again significantly greater in aged mice than in young mice (Figures 1F–H; Mann–Whitney U-test, *n* = 136, 140; median = 15.60, 20.15; IQR = 27.505, 34.75; *p* = 0.0178).

The OSI measures the magnitude of the response difference between neurons in the visual cortex to visual stimuli in the optimal orientation and the orthogonal orientation, reflecting the sensitivity of neurons to stimuli in specific orientations. The OSI was 0.16 lower in the aged group than in the young group (Figure 1I; Mann–Whitney U-test, *n* = 68, 79; median = 0.5836, 0.4207; IQR = 0.5351, 0.4987; *p* = 0.0433). The SNR indicates the difference between the neuron's response to visual stimuli in the optimal orientation and its spontaneous activity. Neurons were classified into two groups based on their evoked firing rate, defined as the difference between the maximum and spontaneous firing rates: >6 and ≤ 6. The SNRs of both neuronal populations were higher in young mice than in aged mice. Specifically, the SNRs of neurons with low evoked firing rates were nearly 0.1 lower in aged mice than in young mice (Figure 1J, right; Mann–Whitney U-test, *n* = 58, 52; median = 0.7025, 0.6225; IQR = 0.1875, 0.2012; *p* = 0.0079). The SNRs of neurons with high evoked firing rates were also slightly lower in aged mice than in young mice (Figure 1J, left; Mann–Whitney U-test, *n* = 78, 88; median = 0.9475, 0.9050; IQR = 0.1331, 0.1788; *p* = 0.0379). The FF is an important measure of neuronal response variability and the fidelity of information transmission, with a higher FF indicating lower fidelity. Determination of the FF revealed that the response variability of single neurons was significantly greater in aged mice than in young mice (Figure 1K; Mann–Whitney U-test, *n* = 136, 140; median = 1.311, 1.498; IQR = 0.765, 1.188; *p* = 0.0232).

These results indicate that neurons in the V1 of aged mice exhibit increased activity compared with those of young mice. Additionally, aged neurons encode sensory stimuli less effectively, as evidenced by a decreased SNR and increased response variability. Moreover, orientation selectivity, one of the most important receptive field properties of visual cortical neurons, is weakened in aged mice. Given that visual information processing in the V1 results from the coordinated activity of interconnected neuronal networks rather than from the function of single neurons in isolation, we next investigated how these cellular alterations impact network-level dynamics and population-wide information processing.

### Aging increases V1 neural network noise levels

**Figure 2:**
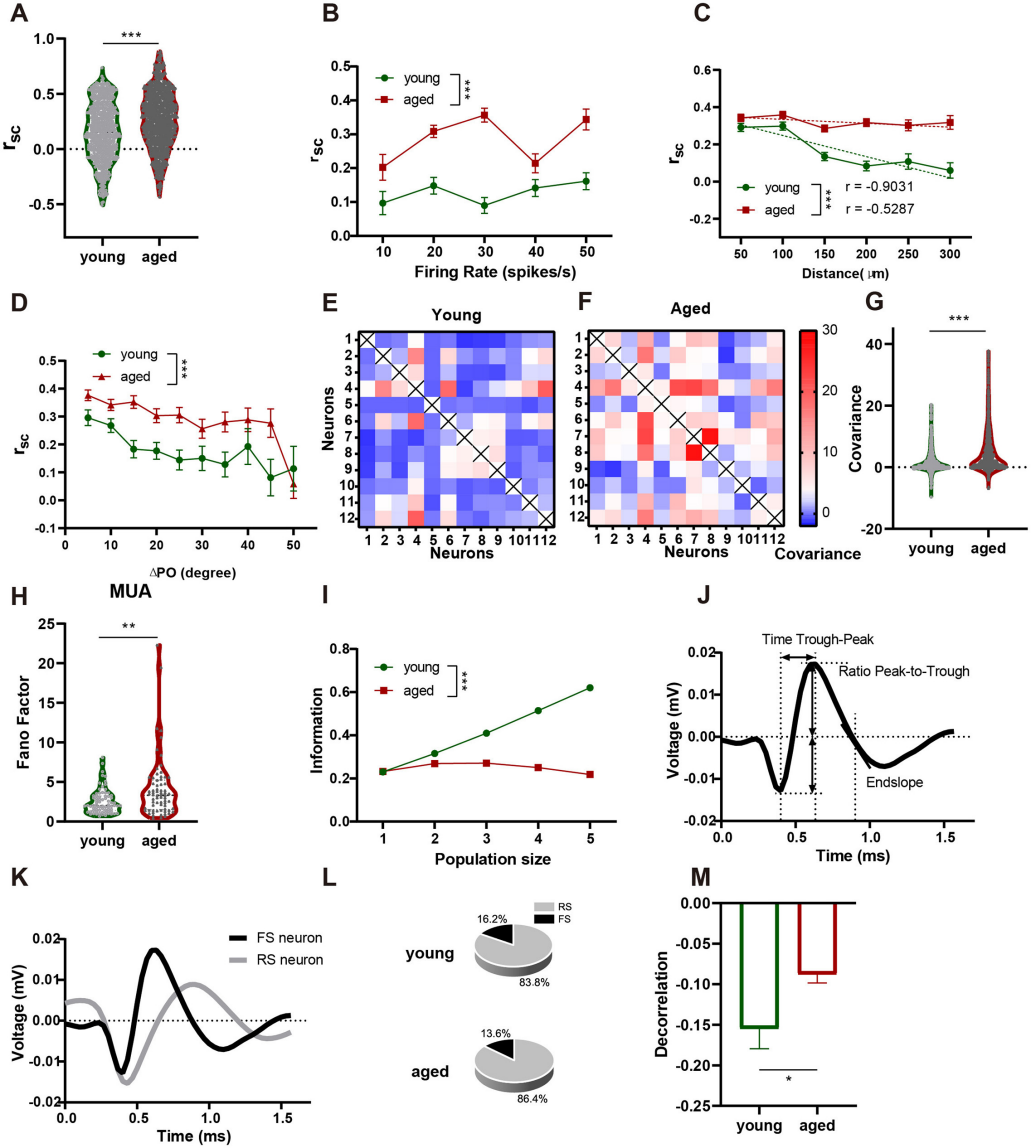
Aging increases V1 neural network noise levels. **(A)** Aging increases the r_sc_ of V1 neurons (Cliff's δ = −0.2305). **(B)** Dependence of *r*_sc_ on firing rate. The firing rate bins started at 0 spikes/s and were increased to 50 spikes/s in increments of 10 spikes/s. The average of all the data were plotted at the maximum value of each bin. Young group, **dots** and **green line**; aged group, **squares** and **red line**. **(C)** Dependence of *r*_sc_ on distance. The distance bins started at 0 μm and increased to 300 μm in increments of 50 μm. The average of all the data were plotted at the maximum value of each bin. **(D)** Covariance of each pair of neurons in the young group. **(E)** Covariance of each pair of neurons in the aged group. **(F)** Aging increases the covariance of V1 neurons (Cliff's δ = −0.3099). **(G)** Aging increases the MUA FF of V1 neurons (Cliff's δ = −0.2747). **(H)** MI varies with the size of the group. **(I)** Example of a representative spike wave of a V1 neuron. The time trough-peak is the duration between the spike trough and peak; the ratio peak-to-trough is the peak/trough amplitude ratio; and the end slope is the slope of the waveform 0.5 ms after the trough. **(J)** Representative FS and RS neurons, which are separated by their spike wave characteristics. FS neurons had a shorter trough-peak duration (<0.26 ms), larger peak-to-trough ratio (>0.8), and negative end slope (−0.05). **(K)** FS neurons classified from the recorded neurons and ISI histograms of representative FS and RS neurons and the ratio of these two types of neurons. **(L)** Aging reduces the decorrelation mediated by V1 inhibitory neurons (Cliff's δ = −0.1417). Data are presented as median and interquartile range in violin plots and mean ± standard error of the mean in **line** and **bar graphs**. ***p < 0.001, **p < 0.01, *p < 0.05. FF, Fano factor; FS, fast spiking; ISI, interspike interval; MI, mutual information; MUA, multi-unit activity; RS, regular spiking; r_sc_, noise correlation; V1, primary visual cortex.

In a local neural network, neighboring neurons receive shared sensory inputs, leading to shared response variability (Azeredo da Silveira and Rieke, 2021). The degree of variability sharing, known as noise correlation, is a key measure of the information-processing capacity of a network (Bartolo et al., 2020; Zohary et al., 1994). Neurons in aged mice exhibited significantly greater noise correlations than those in young mice (Figure 2A; Mann–Whitney U-test, *n* = 709, 1,081, median = 0.1941, 0.3110, IQR = 0.4508, 0.4174; *p* < 0.001). Importantly, *r*_sc_ was significantly higher in the aged group than in the young group at comparable firing rates, suggesting that the observed increase in noise correlation is not solely attributable to elevated firing rates in aged neurons [Figure 2B; two-way ANOVA, *F*_(1, 1, 085)_ = 79.07, *p* < 0.001]. Instead, this result indicates potential age-related changes in underlying network connectivity, such as alterations in the *E*/*I* balance or structural changes in local circuits (Lauterborn et al., 2021; Radulescu et al., 2023; Zhou and Yu, 2018). Additionally, we examined the relationship between noise correlation and the distance between recording sites and found a negative correlation between the two in both the young and aged groups (Figure 2C; young: *r* = −0.9031, aged: *r* = −0.5287). However, compared to the young group, the noise correlation in the aged group was significantly elevated across all distances [Figure 2C; two-way ANOVA, *F*_(1, 1, 670)_ = 95.43, *p* < 0.001]. This suggests that the observed changes in noise correlation cannot be fully explained by differences in the proximity of the recording sites. Noise correlation (*r*_sc_) is influenced by the preferred orientations of neurons, with higher correlations observed between neurons with similar preferred orientations (ΔPO = 0°) and lower correlations as the difference in preferred orientation (ΔPO) increases (Bair et al., 2001; Cohen and Newsome, 2008). To ensure that the observed differences in noise correlation between the aged and young groups were not confounded by differences in preferred orientations, we analyzed the relationship between noise correlation and ΔPO. The results showed that, while noise correlation decreased with increasing ΔPO in both groups, the aged group consistently exhibited significantly higher noise correlations across all ΔPO values compared to the young group. This finding confirms that the age-related increase in noise correlation is independent of differences in preferred orientations [Figure 2D, two-way ANOVA, *F*_[1, 1, 648]_ = 29.35, *p* < 0.001].

Noise correlation reflects shared response variability between neuronal pairs. The increased noise correlation in aged mice suggests changes in the coordination of neuronal activity. To understand how these changes affect population-level dynamics, we analyzed the neuronal response covariance and FF of MUA to measure population noise. Figures 2E, F show examples of the covariance between the 12 neurons recorded simultaneously. The covariance between V1 neurons was significantly higher in aged mice than in young mice (Figure 2G; Mann–Whitney U-test, *n* = 777, 1,067, median = 0.5237; 2.047, IQR = 2.362, 1.5471; *p* < 0.001). Similarly, population response variability, as measured by the FF of MUA, was ~60% greater in aged mice than in young mice (Figure 2H; Mann–Whitney U-test, *n* = 75, 70, median = 2.034, 3.323, IQR = 1.836, 3.714; *p* = 0.0041). These increases in covariance and population noise may indicate a decline in the amount of information encoded by neuronal networks (Averbeck et al., 2006). As shown in Figure 2I, MI plotted as a function of neuronal population size revealed that information content was significantly reduced in aged mice compared with young mice [two-way ANOVA, *n* = 6, 5, *F*_(1, 492, 300)_ = 1,432, *p* < 0.001]. This reduction in MI indicates impaired cortical information processing efficiency, which may contribute to the deficits in behavioral performance observed during aging (Kohn et al., 2016; Nogueira et al., 2020).

Noise correlation is regulated in neuronal networks through a mechanism known as decorrelation, which typically involves inhibitory neurons (Kohn et al., 2016). In *E*/*I* balanced networks, inhibitory signals reduce the impact of shared excitatory inputs on postsynaptic responses, thereby maintaining low levels of noise correlation, a phenomenon referred to as the decorrelation effect (Graupner and Reyes, 2013; Salinas and Sejnowski, 2000). To investigate whether the age-related changes in noise correlation observed in the present study were associated with alterations in decorrelation, we classified neurons into fast-spiking (FS) inhibitory neurons and regular-spiking (RS) excitatory neurons based on their waveform parameters (Figures 2J, K) (Jung et al., 2023; Niell and Stryker, 2008; Wang et al., 2019); FS neurons are predominantly PV^+^-inhibitory neurons (Wamsley and Fishell, 2017). This classification method identified 22 (16.2%) and 19 (13.6 %) FS neurons in the young and aged groups, respectively (Figure 2L, right column). We then analyzed the *E*/*I* neuron pair interactions to quantify decorrelation. The decorrelation effect was significantly weaker in aged mice than in young mice (Figure 2M; Mann–Whitney U-test, *n* = 75, 200, *p* = 0.0452). A more negative decorrelation value represents a stronger decorrelation effect, indicating that inhibitory neurons in aged networks are less effective at reducing noise correlation.

### Metformin improves the function of V1 neurons in aged mice

**Figure 3:**
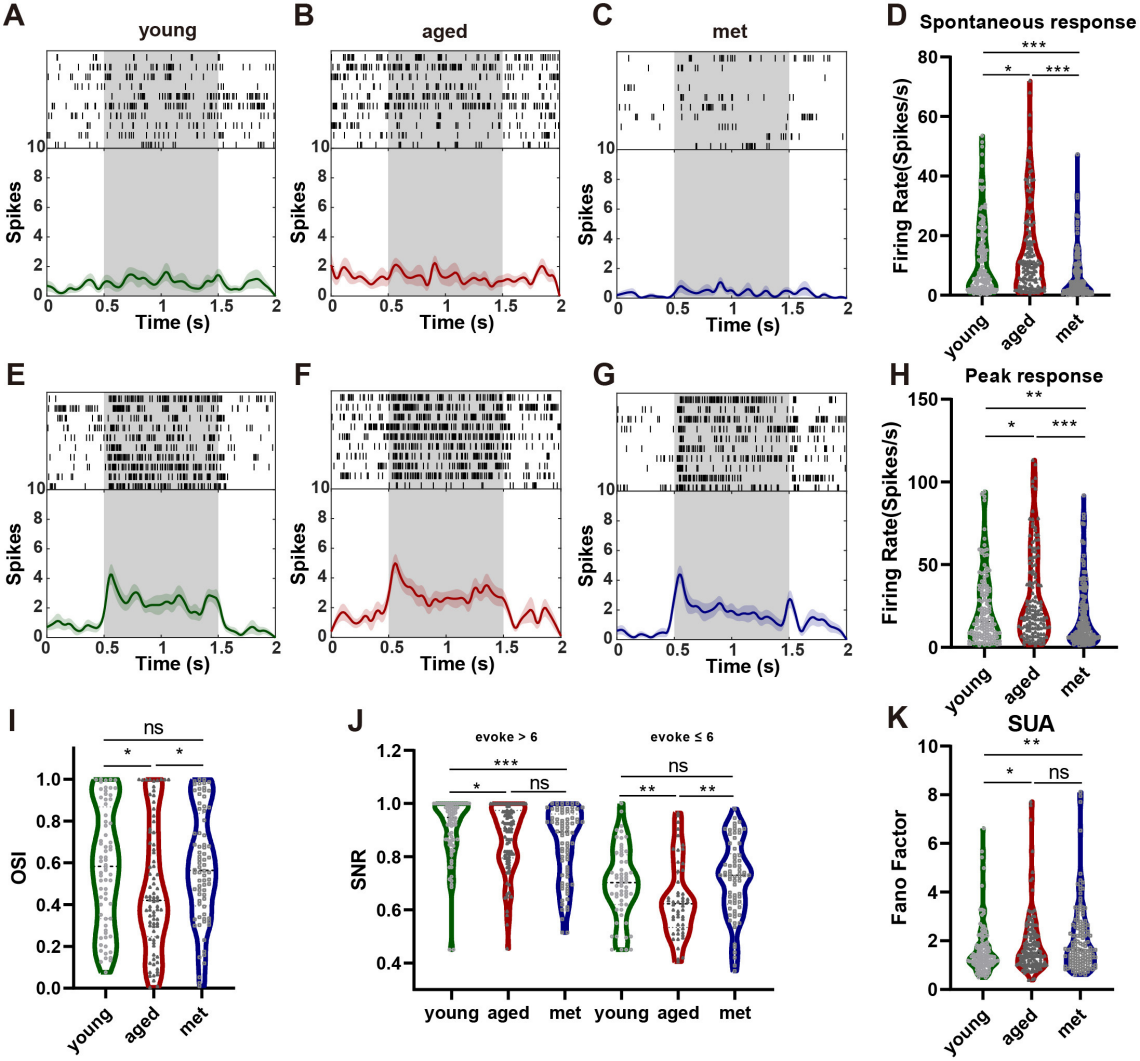
Metformin improves visual cortical neuronal function in aged mice. **(A)** Example of a neuronal spontaneous response in the young group. **(B)**. Example of a neuronal spontaneous response in the aged group. **(C)** Example of a neuronal spontaneous response in the met group. **(D)** Differences in spontaneous responses (aged vs. met: Cliff's δ = 0.4864, young vs. met: Cliff's δ = 0.3349). **(E)** The neuronal peak response to the optimal orientation stimulus in the young group. **(F)** The neuronal peak response to the optimal orientation stimulus in the aged group. **(G)** The neuronal peak response to the optimal orientation stimulus in the met group. **(H)** Differences in peak responses (aged vs. met: Cliff's δ = 0.3347, young vs. met: Cliff's δ = 0.1819). **(I)** Differences in orientation selectivity index (aged vs. met: Cliff's δ = −0.2132, young vs. met: Cliff's δ = −0.0130). **(J)** Differences in SNR of neurons with firing rate >6 (**left**, aged vs. met: Cliff's δ = 0.1201, young vs. met: Cliff's δ = 0.3173) and ≤ 6 (**right**, aged vs. met: Cliff's δ = −0.3266, young vs. met: Cliff's δ = −0.0778). **(K)** Differences in the FF of neurons (aged vs. met: Cliff's δ = −0.0756, young vs. met: Cliff's δ = −0.2288). Data are presented as median and interquartile range in violin plots. ***p < 0.001, **p < 0.01, *p < 0.05. FF, Fano factor; SNR, signal-to-noise ratio.

A total of 146 neurons were recorded and isolated from the seven mice in the met group; 26, 19, 31, 20, 21, 13, and 16 from each mouse. The neuronal activity in met group mice is shown in Figure 3. Spontaneous neuronal firing was significantly lower in the met group than in the aged (Figures 3A–D; Mann–Whitney U-test, *n* = 140, 146; median = 10.45, 2.85; IQR = 16.8, 7.025; *p* < 0.001) and young (Figures 3A–D; Mann–Whitney U-test, *n* = 136, 146, median = 6.739, 2.85, IQR = 16.98, 7.025; *p* < 0.001) groups. When presented with the optimal grating stimulus, the peak neuronal response was also significantly lower in the met group than in the aged (Figures 3E–H; Mann–Whitney U-test; *n* = 140, 146; median = 20.15, 9.8; IQR = 34.75, 19.475; *p* < 0.001) and young (Figures 3E–H; Mann–Whitney U-test; *n* = 136, 146; median = 15.60, 9.80; IQR = 27.505, 19.475; *p* = 0.0083) groups.

The OSI of V1 neurons was significantly greater in the met group than in the aged group (Figure 3I; Mann–Whitney U-test, *n* = 79, 76, median = 0.4207, 0.5642, IQR = 0.4987, 0.4439; *p* = 0.0218), but did not differ significantly between the met and young groups (Figure 3I; Mann–Whitney U-test, *n* = 68, 76, median = 0.5836, 0.5642, IQR = 0.5351, 0.4439; *p* = 0.9952). To assess the ability to extract information from neurons, we determined the SNR in neurons with high and low evoked firing rates. No significant differences were observed between the met and aged groups in terms of the SNR of neurons with high evoked responses (Figure 3J; left; Mann–Whitney U-test, *n* = 88, 76; median = 0.9050, 0.8925; IQR = 0.1788, 0.2275; *p* = 0.1962). The SNR of neurons with low evoked responses was significantly greater in the met group than in the aged group (Figure 3J; right; Mann–Whitney U-test, *n* = 52, 70; median = 0.6225, 0.73; IQR = 0.2012, 0.19; *p* = 0.0020). However, no significant differences in SNR were found between the met and young groups (Figure 3J; right; Mann–Whitney U-test, *n* = 58, 70; median = 0.7025, 0.7300; IQR = 0.1875, 0.19; *p* = 0.4572). Finally, we found no significant difference in FF between the met and aged groups (Figure 3K; Mann–Whitney U-test, *n* = 140, 146, median = 1.498, 1.621; *p* = 0.2689).

### Metformin reduces the level of V1 neural network noise in aged mice

**Figure 4:**
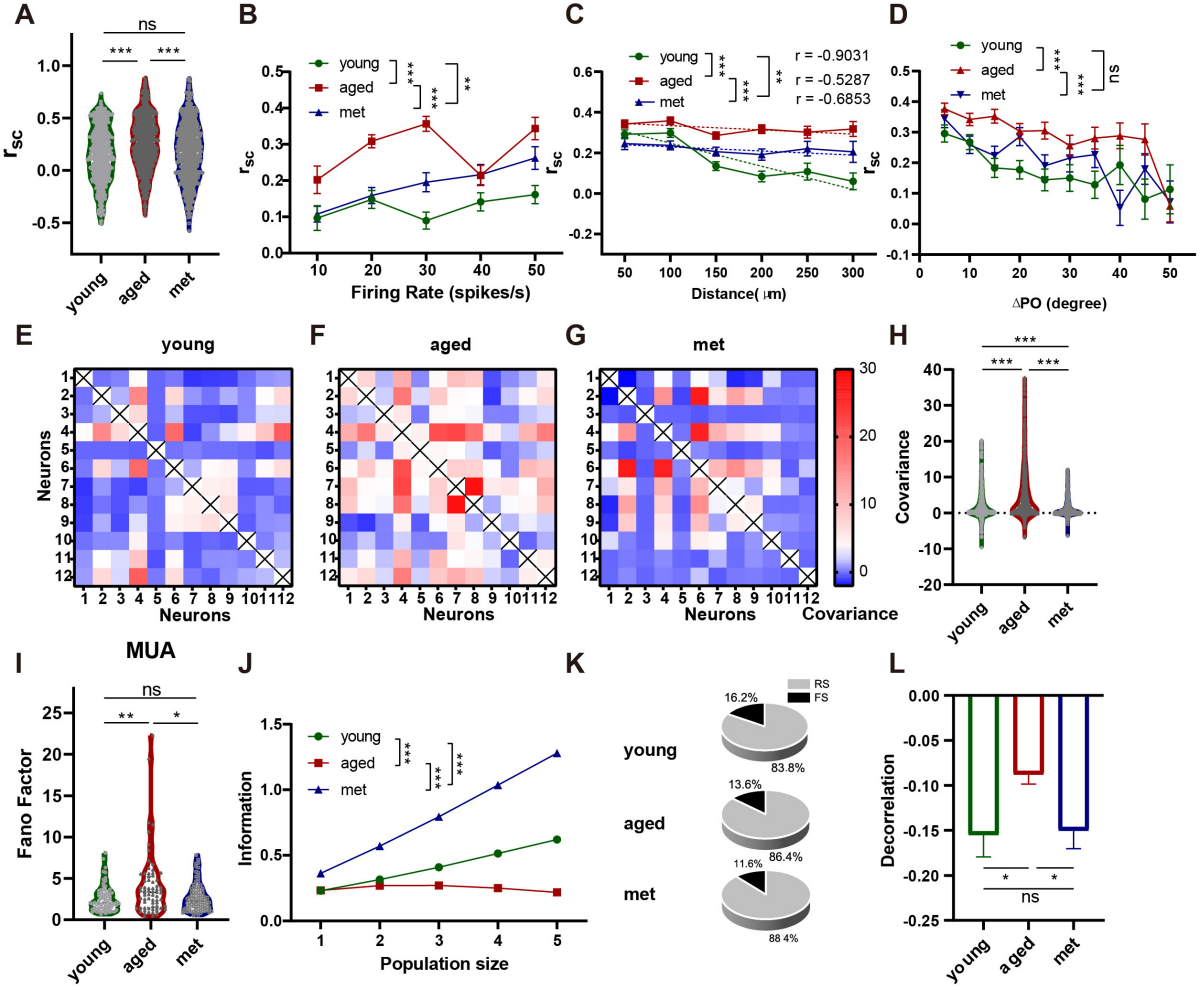
Metformin reduces the level of V1 neural network noise in aged mice. **(A)** Differences in *r*_sc_ (aged vs. met: Cliff's δ = 0.1765, young vs. met: Cliff's δ = −0.0480). **(B)**. Dependence of *r*_sc_ on firing rate. Young group, dots and green line; aged group, squares and red line; met group, triangles and blue line. **(C)** Dependence of *r*_sc_ on distance. **(D)** Covariance of each pair of neurons in the young group. **(E)** Covariance of each pair of neurons in aged group. **(F)** Covariance of each pair of neurons in the met group. **(G)** Differences in covariance (aged vs. met: Cliff's δ = 0.3884, young vs. met: Cliff's δ = 0.0564). **(H)** Differences in the MUA FF of neurons (aged vs. met: Cliff's δ = 0.2417, young vs. met: Cliff's δ = −0.0475). **(I)** MI varies with the size of the group. **(J)** The ratio of the two types of neurons. **(K)** Differences in decorrelation (aged vs. met: Cliff's δ = 0.1244, young vs. met: Cliff's δ = −0.0136). Data are presented as median and interquartile range in violin plots and mean ± standard error of the mean in line and bar graphs. ***p < 0.001, **p < 0.01, *p < 0.05. FF, Fano factor; MI, mutual information; MUA, multi-unit activity; r_sc_, noise correlation; V1, primary visual cortex.

To investigate whether metformin modulates the local neuronal network noise in the V1 of aged mice, noise correlation was compared. The noise correlation in V1 neurons was significantly lower in the met group than in the aged group (Figure 4A; Mann–Whitney U-test, *n* = 1,080, 754, median = 0.3100 vs. 0.2143, IQR = 0.4174, 0.4527; *p* < 0.001). However, no significant difference was observed between the met and young groups (Mann–Whitney U-test, *n* = 709, 754, means = 0.1941 vs. 0.2143, IQR = 0.4508, 0.4527; *p* = 0.5943). Analysis of the relationship between noise correlation and firing rate, as shown in Figure 4B, showed noise correlation decreased significantly in the met group across different firing rates [two-way ANOVA, *F*_(1, 1, 248)_ = 31.88, *p* < 0.001]. Noise correlation and distance between neuron pairs were negatively correlated in the met group (Figure 4C; *r* = −0.6853). Compared to the aged group, noise correlation decreased significantly in the met group [Figure 4C; two-way ANOVA, *F*_(1, 1, 671)_ = 38.96, *p* < 0.001]. Additionally, noise correlation was analyzed against the difference in preferred orientation between neurons. The results showed that metformin reduced noise correlation consistently across all differences in preferred orientation [Figure 4D, two-way ANOVA, *F*_(1, 1, 567)_ = 18.21, *p* < 0.001]. The covariance of neuronal responses was also compared between the met and aged groups (Figures 4E–H), and was significantly lower in the met group than in the aged group (Mann–Whitney U-test, *n* = 1,067, 744, median = 2.047, 0.2934, IQR = 6.1371, 1.5471; *p* < 0.001). Additionally, a slight downward trend was noted compared with that in the young group (Mann–Whitney U-test, n= 777, 744, median = 0.5237, 0.2934, IQR = 6.1371, 1.5471; *p* = 0.0570). The MUA FF of the met group was lower than that of the aged group (Figure 4I; Mann–Whitney U-test, *n* = 70, 93, median = 3.323, 2.225, IQR = 3.714, 2.033; *p* = 0.0117), but not significantly different to that of the young group (Mann–Whitney U-test, *n* = 75, 93, median = 2.034, 2.225, IQR = 1.836, 2.033; *p* = 0.5995). Finally, we found that metformin significantly increased the MI in aged mice [Figure 4J; two-way ANOVA, *n* = 7, *F*_(1, 440, 172)_ = 11,564, *p* < 0.001], improving the ability of the V1 to encode information. This suggests that metformin may modulate network noise by enhancing inhibitory system-mediated decorrelation, and to assess this we classified neurons into FS and RS neurons based on their firing patterns. Seventeen FS neurons were identified, accounting for 11.6% of all the neurons (Figure 4K). We then calculated the decorrelation of FS–RS neuron pairs (Figure 4L), and found a significant increase in decorrelation in the met group compared to the aged group (Mann–Whitney U-test, *n* = 200, 120; *p* = 0.0403), with levels comparable in the met and young groups (Mann–Whitney U-test, *n* = 75, 120; *p* = 0.8659).

### Metformin improves inhibitory system function by regulating GABA expression and inhibitory synapses

**Figure 5:**
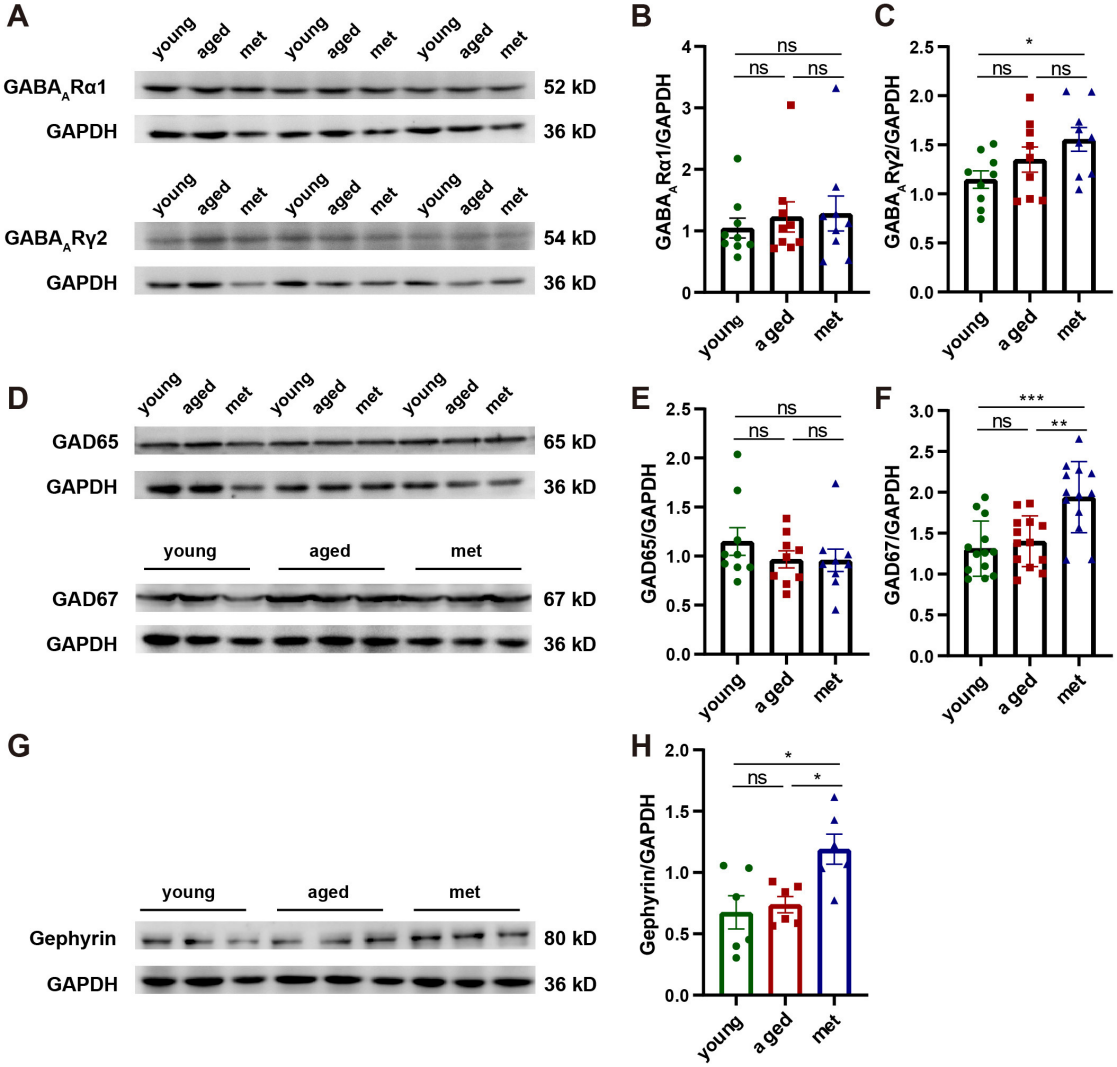
Metformin improves inhibitory system function by regulating GABA expression and inhibitory synapses. **(A)** Metformin did not alter the protein expression levels of GABA_A_Rα1 and GABA_A_Rγ2 subunits in aged mice. **(B)** GABA_A_Rα1 subunit levels (young vs. aged: Cliff's δ = −0.1358, aged vs. met: Cliff's δ = −0.0617, young vs. met: Cliff's δ = −0.1605). **(C)** GABA_A_Rγ2 subunit levels (young vs. aged: Cliff's δ = −0.2840, aged vs. met: Cliff's δ = −0.3827, young vs. met: Cliff's δ = −0.6296). **(D)** Metformin upregulated the protein expression levels of GAD67 but not GAD65 in aged mice. **(E)** GAD65 protein expression levels (young vs. aged: Cliff's δ = 0.2099, aged vs. met: Cliff's δ = 0.0864, young vs. met: Cliff's δ = 0.2346). **(F)** GAD67 protein expression levels (young vs. aged: Cliff's δ = −0.1716, aged vs. met: Cliff's δ = −0.7041, young vs. met: Cliff's δ = −0.7396). **(G)** Metformin upregulated the expression of gephyrin in aged mice. **(H)** Gephyrin protein expression levels (young vs. aged: Cliff's δ = −0.1667, aged vs. met: Cliff's δ = −0.8333, young vs. met: Cliff's δ = −0.7222). Data are presented as mean ± standard error of the mean. ***p < 0.001, **p < 0.01, *p < 0.05.

To investigate the molecular mechanisms underlying the effects of metformin on neuronal and network functions in the visual cortex of aged mice, we quantitatively analyzed the expression levels of GABA receptor signaling-related and GABA synthesis-related proteins in the young, aged, and met groups using western blotting. As shown in Figure 5, GABA_A_Rα1 subunit expression did not differ significantly between the met and aged groups (Figures 5A, B; Mann–Whitney U-test, *n* = 9, 9, *p* = 0.8633). GABA_A_Rγ2 subunit expression was not significantly increased in the met group compared to the aged group (Figures 5A, C; Mann–Whitney U-test, *n* = 9, 9, *p* = 0.1903). However, effect size analysis indicated that there were significant differences between the two groups (Cliff's δ = −0.3827). Compared to the young group, the metformin-treated group showed a significant increase in GABA_A_Rγ2 expression (Mann–Whitney U-test, *n* = 9, 9, *p* = 0.0244, Cliff's δ = −0.6296). We also analyzed the expression of the GABA synthesis-related proteins GAD65 and GAD67 and found that GAD67 expression was significantly higher in the visual cortex of the met group than in the aged group (Figures 5D–F; Mann–Whitney U-test, *n* = 13, 13, *p* = 0.0015). The expression of gephyrin, a protein involved in postsynaptic receptor clustering (Alvarez, 2017), was elevated in the met group compared to the aged group (Figures 5G, H; Mann–Whitney U-test, *n* = 6, 6, *p* = 0.0152), suggesting that metformin regulates postsynaptic receptor aggregation and stabilization, thereby contributing to synaptic transmission. These findings indicate that metformin enhances the inhibitory function of the visual cortex in aged mice by promoting GABA synthesis and release, regulating neurotransmitter–receptor binding, and reducing age-related neuronal hyperactivity.

## Discussion

In the present study, we systematically investigated age-related changes in visual processing and revealed the therapeutic potential of metformin for preserving visual cortical function. Our findings reveal three interconnected layers of age-related alterations in the visual system. At the cellular level, aging induces profound changes in V1 neurons, manifested as increased spontaneous and evoked responses, compromised orientation selectivity, and a deterioration in the SNR. These alterations are accompanied by network-level dysfunctions, such as elevated noise correlation and enhanced response covariance in neuronal populations, which reflect impaired coordination of neuronal activity and reduced information-processing efficiency. Notably, daily metformin administration effectively ameliorated these age-related changes through a mechanism involving enhanced inhibitory signaling and reduced network noise, suggesting a promising therapeutic strategy for age-related visual decline.

Our results demonstrated that the ability of visual cortical neurons to process visual information declines significantly in aged animals. These findings are consistent with those of previous studies on monkeys and cats (Hua et al., 2006; Schmolesky et al., 2000; Yu et al., 2006). In addition, we observed that the interactions among neuronal populations and their capacity to process noise in the primary visual cortex deteriorated in aged mice. This aligns with our earlier observations in aged rhesus monkeys (Wang et al., 2019), further supporting the notion that impaired noise processing by neuronal populations is a key characteristic of visual cortical aging.

The observed increase in *r*_sc_ in the V1 of aged mice provides important insights into age-related changes in neural network function. Noise correlation is a critical measure of the shared variability among neurons and directly reflects the degree of functional coupling within neural populations. In our study, the aged group exhibited significantly higher noise correlations than the young group, even when the firing rates were matched, which implies the presence of intrinsic changes in network connectivity or dynamics. Previous studies have shown that noise correlation is influenced by the *E*/*I* balance (Cohen and Kohn, 2011; Renart et al., 2010). Therefore, the elevated *r*_sc_ in aged mice may reflect a disruption in this balance, potentially due to a decline in inhibitory function or the structural remodeling of local circuits (Leventhal et al., 2003; Schmolesky et al., 2000).

Additionally, the relationship between noise correlation and the distance between recording sites revealed that the noise correlation in aged mice remained elevated across all distances. In young mice, noise correlation decreased with increasing distance, consistent with the predominance of short-range horizontal connections in the V1 (Ts'o et al., 1986). However, the reduced dependence on distance in aged mice suggests a breakdown in this spatial organization, potentially due to aberrant long-range connectivity or a loss of specificity in local network interactions. This finding aligns with previous reports of increased neural noise and reduced precision in aging cortical circuits (Gao and Penzes, 2015). Furthermore, this change in the distance—*r*_sc_ relationship could indicate a shift toward more globally synchronized activity in the neuronal network, which may impair the ability of the cortical network to segregate information spatially and temporally.

Collectively, these results suggest that aging is associated with a fundamental reorganization of neural population dynamics in the visual cortex. The elevated noise correlation, at matched firing rates and across all distances, points to both local and global changes in network connectivity and functional coupling. These alterations may diminish the capacity of the network to efficiently encode and process sensory information, contributing to the age-related decline in visual perception (Betts et al., 2007).

MI reflects the amount of information shared between neuronal responses and external stimuli, and serves as a key measure of the ability of the network to reliably process sensory inputs (Kohn et al., 2016). The observed reduction in MI in the aged group in the present study therefore indicates a significant decline in the efficiency of information encoding and transmission within neuronal networks, and suggests that age-related increases in noise correlation and population variability impair the precision of neural coding. Elevated noise correlation limits the independence of neuronal responses, thereby reducing the diversity of encoded information (Averbeck et al., 2006). Additionally, the increased FF indicates heightened response variability, which may further compromise information encoding. From a functional perspective, the decrease in MI likely reflects disruptions in cortical network dynamics, such as impairments in the *E*/*I* balance or changes in synaptic plasticity (Leventhal et al., 2003; McQuail et al., 2015). These network-level changes in information-processing efficiency may underlie age-related declines in sensory perception, decision making, and motor control (Nogueira et al., 2020).

Future studies should explore the specific circuit mechanisms underlying these changes, such as alterations in inhibitory interneuron function, long-range connectivity, and synaptic noise. Interventions targeting these mechanisms could represent potential strategies to mitigate the age-related decline in cortical information processing and associated behavioral impairments.

In our study, elevated spontaneous and evoked neuronal responses in the visual cortex of aged animals were accompanied by a decline in orientation selectivity. These findings suggest a potential mechanism by which aging leads to the impairment of the inhibitory system. Orientation selectivity in the visual cortex is the result of a combination of feedforward inputs and intracortical inhibitory regulation (Balla et al., 2025; Rossi et al., 2020). The decline in inhibitory function is reflected in reduced orientation tuning of neurons (Leventhal et al., 2003). Our decorrelation analysis revealed that the decorrelation effect mediated by FS neurons was significantly impaired in aged mice, which may contribute to increased noise correlations within the neural network. However, this conclusion is based previous studies (Barta and Kostal, 2024; Graupner and Reyes, 2013), as we did not directly manipulate FS neurons or inhibitory transmission. This disruption could potentially limit the ability of the network to integrate information from single neurons in a weighted manner, thereby reducing the efficiency of population signal aggregation (Shadlen and Newsome, 1998). Future studies employing techniques such as optogenetics or chemogenetics will be essential to establish the causal relationship between impaired FS neuron function and changes in network dynamics. Using waveform analysis, we identified weakened decorrelation effects in FS neurons, which are predominantly PV^+^-inhibitory neurons. This finding highlights the age-related deterioration of the GABAergic inhibitory system and its critical role in regulating noise correlations and maintaining network functions. The increased noise correlation observed in the visual cortex of aged mice provides further evidence for the age-related decline in inhibitory function.

Metformin administration mitigated these age-related impairments. Metformin treatment upregulated GAD67 and gephyrin protein expression levels, reduced spontaneous and evoked neuronal responses, and improved orientation selectivity and the SNR. At the network level, metformin treatment reduced noise correlation and population response variability, leading to an increase in the amount of visual information encoded by the network. These findings suggest that metformin-induced improvements in the GABAergic inhibitory system can effectively counteract the age-related functional decline of the visual cortex.

Western blot analysis revealed that metformin exerted a specific effect on GABAergic signaling, selectively upregulating GAD67 and gephyrin expression, indicating enhanced presynaptic GABA synthesis and postsynaptic organization. Notably, metformin did not significantly alter the expression of GABA_A_ receptor subunits, suggesting that its primary effect lies in optimizing synaptic efficiency. The upregulation of gephyrin, a scaffolding protein critical for organizing and stabilizing inhibitory synapses (Alvarez, 2017), may explain the observed improvements in neuronal response properties and network dynamics.

Despite the significant effects of metformin in the amelioration of age-related visual cortical dysfunction, several important questions remain unanswered. The diverse inhibitory neurons in the visual cortex may play distinct roles in visual processing and exhibit varying susceptibilities to aging due to differences in morphology, structure, cortical distribution, and connectivity (DeFelipe, 1993; Markram et al., 2004; Tremblay et al., 2016). A previous study showed that metformin preferentially enhances glutamatergic rather than GABAergic transmission in the hippocampal CA1 region (Chen et al., 2020). This difference may be explained by variations in exposure duration, dosage, and experimental approaches, as we used a relatively high dose of metformin for 3 weeks, whereas Chen et al. exposed brain slices to lower concentrations of metformin (1 and 10 μM) *in vitro*. Furthermore, the mechanisms of action of metformin may vary across brain regions based on structural and functional demands or dosage. These findings also suggest that the modulation of the *E*/*I* balance by metformin may not be unidirectional. Understanding the temporal dynamics of the therapeutic effects of metformin and their persistence after treatment discontinuation may provide critical insights for the development of clinical strategies. Our findings also raise intriguing possibilities for the broader application of metformin in age-related sensory decline, as the mechanisms identified here may extend to other sensory systems affected by aging.

## Data Availability

The original contributions presented in the study are included in the article/supplementary material, further inquiries can be directed to the corresponding author/s.
